# Effects of Licorice Functional Components Intakes on Blood Pressure: A Systematic Review with Meta-Analysis and NETWORK Toxicology

**DOI:** 10.3390/nu16213768

**Published:** 2024-11-02

**Authors:** Tianyu Wu, Jingyi Yang, Jiayue Xia, Guiju Sun

**Affiliations:** Key Laboratory of Environmental Medicine and Engineering of Ministry of Education, Department of Nutrition and Food Hygiene, School of Public Health, Southeast University, Nanjing 210009, China; tianyu_w@seu.edu.cn (T.W.); yjy_1063@163.com (J.Y.); 230229010@seu.edu.cn (J.X.)

**Keywords:** licorice, meta-analysis, hypertension biomarkers, machine learning, network toxicology, molecular docking

## Abstract

Objective: To investigate the effects of licorice functional ingredient intake on blood pressure, explore its potential mechanisms of action, and provide safety information for personalized nutritional interventions in special populations and for the application of licorice-derived functional foods. Methods: PubMed, Cochrane Library, Medline, Embase, EBSCO, ScienceDirect, and Web of Science databases were searched from inception to 31 August 2024. Randomized controlled trials (RCTs) investigating the intake of licorice or its functional components were included. The range of continuous variables was assessed using the weighted mean difference (WMD) with 95% confidence intervals. Genes associated with hypertension were screened using an online database. Machine learning, receiver operating characteristic(ROC) curve analysis, molecular docking, and gene set enrichment analysis (GSEA) were employed to explore the potential mechanisms underlying licorice-induced blood pressure fluctuations. Results: Eight RCTs (541 participants) were included in the meta-analysis, which indicated interventions containing glycyrrhizic acid (GA) as the main component increased systolic blood pressure (SBP) and diastolic blood pressure (DBP) (SBP: WMD [95% *CI*] = 3.48 [2.74, 4.21], *p* < 0.001; DBP: WMD [95% *CI*] = 1.27 [0.76, 1.78], *p* < 0.001). However, interventions dominated by licorice flavonoids(LF) had no significant effect on SBP or DBP (SBP: WMD [95% *CI*] = 0.58 [−1.15, 2.31], *p* = 0.511; DBP: WMD [95% *CI*] = 0.17 [−1.53, 1.88], *p* = 0.843). Three machine learning algorithms identified five biomarkers associated with hypertension: calmodulin 3 (CALM3), cluster of differentiation 9 (CD9), growth factor independence 1B transcriptional repressor (GFI1B), myosin light chain kinase (MYLK), and Ras suppressor-1 (RSU1). After removing biomarkers with lower validity and reliability, GFI1B, MYLK, and RSU1 were selected for subsequent analysis. The network toxicology results suggested that GA and its metabolite glycyrrhetinic acid may act on GFI1B, MYLK, and RSU1, influencing blood pressure fluctuations by modulating nitrogen metabolism signaling pathways. Conclusions: There were distinct differences in the effects of licorice functional components on blood pressure. Functional constituents dominated by GA were shown to increase both SBP and DBP, whereas those dominated by LF did not exhibit significant effects on blood pressure. The hypertensive mechanism of GA may involve the modulation of GFI1B, MYLK, and RSU1 to regulate nitrogen metabolic pathways.

## 1. Introduction

There has been a focus in recent years on the concept of food nutrition as medicine [1]. Medicine and food homology substance have the ability to provide an organism with a wealth of nutrients, and can also prevent or be a secondary treatment for some chronic diseases [2,3].Licorice is classified as a medicine and food homology substance in China and it has a wide range of uses in the fields of food supplements and pharmaceutical applications [4]. Due to its diverse bioactive components, licorice plays a critical role in the management of gastrointestinal, respiratory, and liver diseases, as well as serving as an adjunct therapy in drug treatment [5]. Licorice and its extracts have been extensively utilized in traditional Chinese and European medicine, contributing to a global market value exceeding several million dollars annually [6,7]. Kong J et al. demonstrated that licorice can improve cellular autophagy and reduce inflammatory responses in the treatment of ulcerative colitis through its anti-inflammatory and antioxidant properties [8]. Wang M et al. found that the active components of licorice alleviate alcoholic fatty liver disease by reducing oxidative stress [9]. Additionally, as a traditional herb, licorice plays a significant role in various traditional Chinese medicine formulations [10,11].Glycyrrhizic acid (GA), the primary triterpenoid saponin in glycyrrhiza glabra, is extensively used as a food additive in the processing of foods and tobacco [12]. Research has demonstrated the therapeutic potential of GA in treating hepatitis, neurological disorders, and ischemic brain injury [13]. The flavonoid constituents of glycyrrhiza glabra, including glycyrrhizin, isoglycyrrhizin, and photoglycyrrhizidine, exhibit anti-inflammatory, antioxidant, and immunomodulatory properties, making licorice flavonoids(LF) valuable functional ingredients in nutraceuticals [7]. Despite its broad use in functional foods and pharmaceuticals, the safety of licorice remains a concern, particularly in populations with underlying cardiovascular conditions [14].

Numerous studies have reported adverse effects associated with licorice consumption, such as hypokalemia, hypertension, and metabolic alkalosis [15]. It has been proposed that licorice or its active components inhibit 11β-hydroxysteroid dehydrogenase, which blocks cortisol conversion and increases mineralocorticoid levels, leading to pseudoaldosteronism. This disruption of cortisol and aldosterone pathways results in electrolyte imbalances, ultimately affecting systolic blood pressure (SBP) and diastolic blood pressure (DBP) [16]. Interestingly, licorice-induced adverse effects are highly dose-dependent and influenced by individual genetic susceptibility [17,18,19]. Moreover, chronic low-dose consumption of licorice in processed foods may induce subclinical effects, warranting further investigation into long-term exposure [20]. Elevated blood pressure is strongly linked to increased arterial disease burden, target organ damage, adverse cardiovascular and cerebrovascular events, and mortality [21]. In hypertensive patients, fluctuations in blood pressure may cause more severe organ damage. While GA and its metabolites are commonly believed to be the main contributors to licorice-induced hypertension, some population-based studies suggest that GA does not consistently elevate blood pressure [19,22]. Therefore, further research is required to identify the specific licorice components responsible for blood pressure changes, with the goal of informing nutritional interventions for vulnerable populations and the clinical use of licorice-derived compounds.

**Figure 1:**
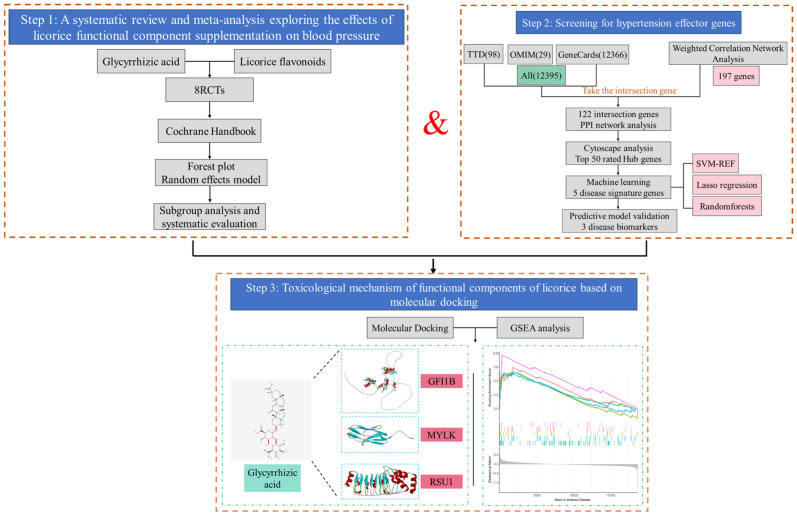
Research scheme. Step 1: We conducted a comprehensive search across multiple databases for RCTs on licorice and blood pressure. Relevant studies were classified into two groups based on the primary licorice components, and duplicates were removed. Articles were then screened by abstract, with eight studies ultimately included. The publication bias of the included studies was assessed using the RevMan tool, and a quantitative analysis was performed using Stata. Step 2: Hypertension-associated genes were identified from TTD, OMIM, and GeneCards databases, complemented by transcriptomic data to screen additional hypertension-related genes. The intersection of these genes was further refined to identify hypertension biomarkers using machine learning methods. Step 3: Step 1 revealed a blood pressure-elevating effect of GA; therefore, toxicology prediction, molecular docking, and GSEA were employed to explore the potential mechanisms underlying this effect.

This study focuses on the impact of GA- and LF-based interventions on SBP and DBP. Moreover, we employed three machine learning algorithms to identify key effector genes associated with hypertension by using network toxicology, transcriptomic datasets, and multiple online databases. Molecular docking and gene function enrichment analysis were conducted to investigate the binding interactions and potential mechanisms of licorice constituents on these hypertension-related genes. The aim of this study was to elucidate the effects of licorice functional ingredients on blood pressure fluctuations and to provide safety dietary recommendations for their use in functional foods. The research scheme of this study is shown in Figure 1.

## 2. Materials and Methods

### 2.1. Search Strategy and Inclusion and Exclusion Criteria

This systematic review and meta-analysis were registered in PROSPERO (Registration No. CRD42024588113). A comprehensive search was conducted across multiple databases, including PubMed, Cochrane Library, Medline, Embase, EBSCO, ScienceDirect, and Web of Science, covering the period from database inception to 31 August 2024. We included RCTs assessing the intake of licorice or its functional components. Both subject-specific and free-text search strategies were employed. The search terms of the study and the number of articles searched in each database were shown in Supplementary Table S1. Following the database search, the relevant literature was reviewed to identify any additional studies potentially overlooked. The literature screening adhered to PRISMA guidelines, and data extraction followed a thorough examination of the full texts. Two independent researchers conducted the literature collection and screening, with disagreements resolved by a third researcher.

The inclusion criteria encompassed population-controlled studies that investigated the oral intake of interventions containing licorice or licorice components. Since GA is the primary compound in licorice, accounting for 8.2 ± 4.1% of the dried herb, with a higher concentration than LF (2.8 ± 1.5%), and given its more evident hypertensive effect [5], studies involving the consumption of licorice (excluding ethanol extracts of licorice) were categorized under GA-related research. 

The exclusion criteria were as follows: (1) human trials without a control group or involving concurrent use of other drugs; (2) trials lacking baseline or post-intervention data; and (3) population-based trials without a conventional control group, observational studies, case reports, reviews, abstracts without full texts, and animal studies.

### 2.2. Data Extraction and Risk of Bias Assessment

For each included study, data were summarized on authorship, publication year, country, methodology, sample size (control and intervention groups), gender distribution, intervention duration, age, dosage, and outcome variables. Two independent researchers extracted the data and conducted a descriptive review of the outcomes, focusing on intervention types, population characteristics, and results.

The risk of bias was assessed using the Cochrane Risk of Bias Tool [23], which evaluates seven domains: random sequence generation, allocation concealment, participant and staff blinding, blinding of outcome assessment, completeness of outcome data, selective reporting, and other biases. Study quality was categorized as high risk, unclear risk, or low risk of bias. Discrepancies between researchers were resolved through the involvement of a third researcher. The risk of bias assessment results were visualized using Review Manager 5.4 software.

### 2.3. Network Toxicology to Explore the Potential Mechanisms by Which Functional Components of Licorice Affecting Blood Pressure

#### 2.3.1. Network Toxicology Predicts Toxicological Effects of GA

The organ-specific toxicity and toxicological endpoints of GA were investigated using the Pro Tox-3.0 database (https://tox.charite.de/protox3/, 30 September 2024), which utilizes molecular similarity, drug moieties, and other features to enhance the accuracy of its predictions through multiple machine learning models [24]. By inputting “glycyrrhizic acid” into the platform, we examined the compound’s properties, oral toxicity classification, and target organs associated with toxicity. The analysis provides high-confidence predictions of GA’s toxicological outcomes.

#### 2.3.2. Hypertension-Related Gene Acquisition

Hypertension-related genes were retrieved from the GeneCards (https://www.genecards.org/, 31 August 2024), Online Mendelian Inheritance in Man (OMIM, https://www.omim.org/, 31 August 2024), and Therapeutic Target Database (TTD, https://db.idrblab.net/ttd/, 31 August 2024) databases using “hypertension” as the search term, with a relevance score threshold of ≥ 10 on GeneCards. Duplicates were removed, and the targets from the three databases were integrated.

The GSE71994 dataset from the GEO database was used to further refine the selection of hypertension-related genes. Transcriptome data were normalized using the “normalizeBetweenArrays” method and analyzed with the limma package for differential expression, with the parameters set to *p* < 0.05 and |log2 fold change| ≥ 1 [25]. The top 5000 genes based on *p*-values were selected for weighted correlation network analysis (WGCNA). The best power value was chosen when the signed R^2^ = 0.85 to construct the co-expression network [26]. Module genes that had the highest correlation with hypertension traits and statistically significant *p*-values were considered hypertension-related genes. The intersection of the genes identified by both methods was obtained using the Venn package in R 4.3.2 software.

#### 2.3.3. Construction of Protein Interaction Networks and Screening of Potential Effector Genes

The hypertension-related genes were entered into the STRING database to construct a protein–protein interaction (PPI) network, restricting species to “Homo sapiens” and setting the interaction confidence threshold to 0.400. The PPI network was visualized using Cytoscape 3.8.1 software, and key nodes were identified and ranked using the CytoHubba plugin. The top 50 genes, ranked by node degree, were selected as potential hypertension effector genes.

#### 2.3.4. Screening and Expression Profiling of Key Hypertension Biomarkers

Among the 50 potential effector genes, key biomarkers were identified using three machine learning algorithms via R 4.3.2 software: SVM-REF, Lasso regression, and Random Forests. The SVM-REF algorithm applied ten-fold cross-validation to rank genes, selecting the top-ranked ones based on accuracy. Lasso regression and Random Forests were similarly applied, and the top-ranked genes were selected as hypertension signature biomarkers. The intersection of the results from the three methods was taken as the final set of biomarkers. Logistic regression models and receiver operating characteristic (ROC) curves were constructed to verify the accuracy of these biomarkers. An area under the ROC curve (AUC) of less than 0.7 is considered low, 0.7–0.9 is considered moderate, and greater than 0.9 is considered high [27]. Additionally, the gene expression was explored in the GSE71994 dataset using R 4.3.2.

#### 2.3.5. Molecular Docking and Single Gene Set Enrichment Analysis (GSEA) Analysis

The interaction between GA and hypertension biomarkers was explored based on findings from the meta-analysis. The 2D structures of GA and glycyrrhetinic acid were obtained from PubChem (https://pubchem.ncbi.nlm.nih.gov/, 31 August 2024), and its 3D structure was optimized using ChemDraw. Protein receptors were identified from UniProt (https://www.uniprot.org/, 31 August 2024), and their 3D structures were downloaded from AlphaFold (https://alphafold.com/, 31 August 2024) and the Protein Data Bank (PDB, https://www.rcsb.org/, 31 August 2024). AutoDockTools 1.5.7 was used to analyze the active sites of the protein receptors, and the binding energies of the small molecules were calculated and visualized using PyMOL and Discovery Studio 2019.

GSEA was performed using R 4.3.2 software. Effector biomarker expression was divided into high and low groups, followed by differential expression analysis using the limma package. LogFC values were extracted and converted into gene IDs to generate a ranked gene list. GSEA based on the KEGG pathway database was performed to explore the mechanisms of action of licorice’s functional components. 

### 2.4. Statistical Analysis

Data were integrated using Excel to extract the number of patients, differences in outcome means, and differences in standard deviation (SD) values for the intervention and control groups. The data were statistically analyzed using STATA 18.0 software and the random effects model was used to calculate the weighted mean difference (WMD) between the mean values of SBP and DBP at the end of the trial in the control and intervention groups to assess the effect of the functional components of licorice on blood pressure. Further, 95% confidence intervals (95% *CI*) for the increase in blood pressure were illustrated by forest plots. *I*^2^ was used to measure the heterogeneity of the study. *I*^2^ values of 25%, 50%, and 75% were considered to represent low, moderate, and high levels of heterogeneity. Subgroup analyses were performed based on the duration of intervention (< or ≥8 weeks), the dosage of GA-based interventions (< or ≥100 mg/day), and the dose of LF-based interventions (< or ≥300 mg/day). Funnel plots and meta-regression analyses were performed to assess the potential impact of publication bias on this study.

*T*-test was used for comparisons between groups. Differences between groups were compared using the built-in function t.test() in the R software. The *p*-value < 0.05 was considered statistically significant.

## 3. Results

### 3.1. Basic Information on the Included Literature

**Figure 2:**
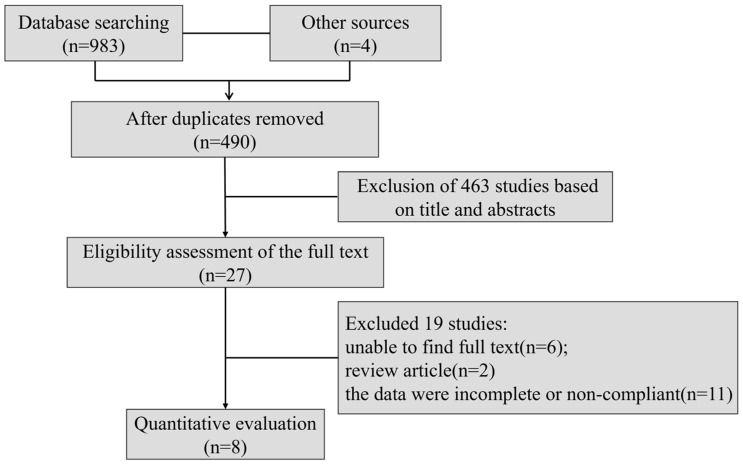
Study screening flowchart.

A total of 987 papers were searched and 27 papers were selected for assessment after reading the titles and abstracts. Nineteen of these were excluded because the full text could not be found (*n* = 6), the data were incomplete or non-compliant (*n* = 11), or they were review articles (*n* = 2), resulting in the inclusion of eight RCTs (Figure 2). Of the eight studies, the trial by CEM van Gelderen et al. was categorized into three studies depending on the dose of the interventions [28]; the study by Ominag et al. was also categorized into five studies depending on the dose of the interventions [29,30]; the study by Bell et al. was categorized into two studies depending on the intervention population selected [31]. In summary, 14 studies were quantitatively analyzed in this study [20,28,29,30,31,32,33,34].

### 3.2. Effects of Licorice Functional Components on Blood Pressure

**Figure 3:**
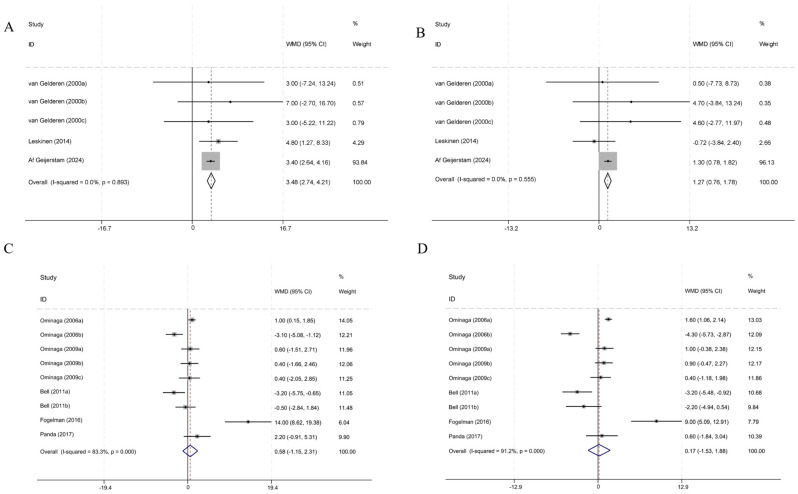
Forest plot of weight difference and 95% confidence intervals for the effect of GA or LF intake on SBP and DBP. (**A**,**B**) WMD and 95% CI of the effects of GA-based interventions on SBP and DBP. (**C**,**D**) WMD and 95% CI of the effects of LF-based interventions on SBP and DBP [20,28,29,30,31,32,33,34].

Five RCTs involving GA as the intervention (74 participants in the control group and 63 in the intervention group) used SBP and DBP as outcome variables. Compared to the control group, the intake of GA as the primary intervention resulted in an increase in SBP (Figure 3A) and DBP (Figure 3B) (SBP: WMD [95% *CI*] = 3.48 [2.74, 4.21], *p* < 0.001; DBP: WMD [95% *CI*] = 1.27 [0.76, 1.78], *p* < 0.001). Both studies showed low heterogeneity (SBP: *I*^2^ = 0.00, *p* = 0.893; DBP: *I*^2^ = 0.00, *p* = 0.555). Information on all the literature on GA can be found in Supplementary Table S2.

Nine RCTs involving LF as the primary licorice intervention (218 participants in the control group and 233 in the intervention group) found no significant effect on SBP (Figure 3C) or DBP (Figure 3D) when compared to the control group (SBP: WMD [95% *CI*] = 0.58 [−1.15, 2.31], *p* = 0.511; DBP: WMD [95% *CI*] = 0.17 [−1.53, 1.88], *p* = 0.843). Both studies demonstrated substantial heterogeneity (SBP: *I*^2^ = 83.3%, *p* < 0.001; DBP: *I*^2^ = 91.2%, *p* < 0.001). 

### 3.3. Risk of Publication Bias and Subgroup Analysis

The results of the publication risk of bias assessment for the included studies were summarized in Supplementary Figure S1. The included studies were assessed for bias using the Cochrane Handbook. Although all of the included studies were RCTs, only two papers provided methods for generating randomized sequences. Some studies did not specify the allocation scheme for subjects, and differences were noted in the findings. As a result, several studies in the areas of allocation concealment, participant and staff blinding, and blinding during outcome assessment were classified as unclear.

**Figure 4:**
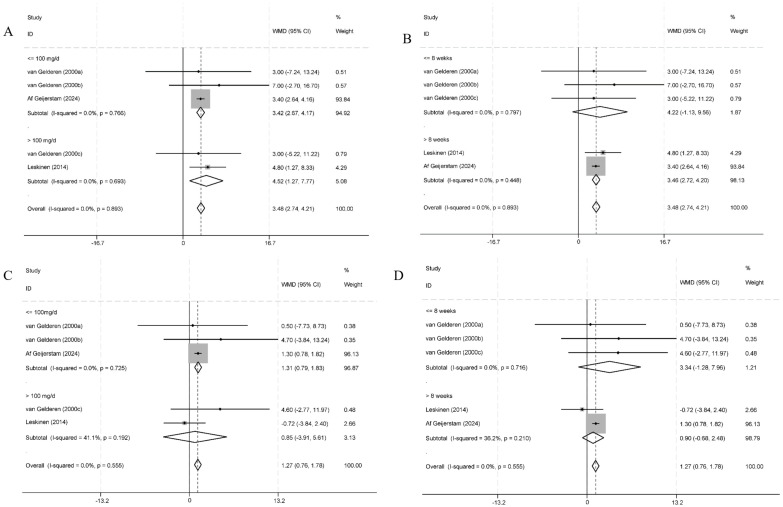
Subgroup analysis of the effect of GA-based interventions on blood pressure. (**A**,**B**) Effect of GA-based interventions intake on SBP at different doses and intervention times. (**C**,**D**) Effect of GA-based interventions intake on DBP at different doses and intervention times [20,28,32].

Subgroup analysis was performed based on the dosage of GA intake and the duration of the intervention, derived from data extracted from the literature. The analysis indicated a significant correlation between the intake of GA and the duration of intervention, and abnormal elevations in SBP. A GA intake of more than 100 mg/day had a greater effect on SBP compared to an intake of ≤100 mg/day (*p* < 0.001) (Figure 4A). Similarly, intervention durations of ≥8 weeks had a greater impact on SBP than durations of less than 8 weeks (*p* < 0.001) (Figure 4B). In terms of DBP, GA intake of ≤100 mg/day was associated with an increase in DBP (*p* < 0.001), while intake of more than 100 mg/day had no significant effect on DBP (*p* = 0.726) (Figure 4C). Moreover, the intervention duration had no significant effect (*p* > 0.05) (Figure 4D). Due to the high heterogeneity observed in the meta-analysis results of trials involving LF, no subgroup analysis was conducted for the compounds. The sources of heterogeneity for LF-related studies were investigated through meta-regression analysis. However, the results did not indicate that intervention duration and dose were potential factors contributing to the heterogeneity (Supplementary Table S3-1 and Supplementary Table S3-2). Information on all the literature about LF can be found in Supplementary Table S3-3. 

### 3.4. Potential Toxic Effects of GA

**Figure 5:**
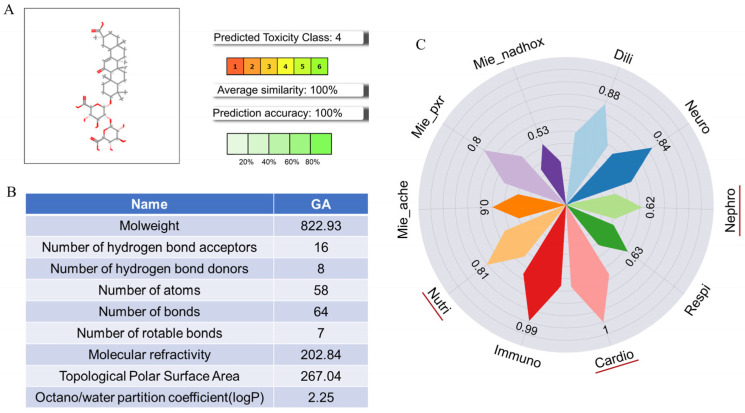
Toxicological prediction results based on Pro Tox-3.0. (**A**) Oral toxic effect class of GA. (**B**) Basic chemical properties of GA. (**C**) Prediction of toxic effects of GA. Dili: drug-induced liver injury; Neuro: neurotoxicity; Nephro: nephrotoxicity; Respi: respiratory toxicity; Cardio: cardiotoxicity; Immuno: immunotoxicity; Nutri: nutritional toxicity; Mie_ache: achetylcholinesterase; Mie_pxr: pregnane X receptor; Mie_nadhox: NADH-quinone oxidoreductase.

The Pro Tox-3.0 prediction results indicate that GA had an oral toxicity classification of level 4, suggesting potential toxicity (Figure 5A). The molecular weight of GA was 822.93 g/mol, with 16 hydrogen bond donors, 8 hydrogen bond acceptors, a log *p* of 2.25, and a topological polar surface area of 267.04 (Figure 5B). These findings suggest that GA had poor bioavailability and might be difficult to metabolize in vivo. Regarding its toxicological effects, we identified several potential oral toxicity endpoints for GA (Figure 5C): nephrotoxicity (probability = 0.62), respiratory toxicity (probability = 0.63), cardiotoxicity (probability = 1.00), immunotoxicity (probability = 0.99), nutritional toxicity (probability = 0.81), acetylcholinesterase inhibition (probability = 0.60), pregnane X receptor modulation (probability = 0.80), and NADH-quinone oxidoreductase inhibition (probability = 0.53); hepatotoxicity and neurotoxicity of GA were not observed in the adverse outcome endpoints: drug-induced liver injury (probability = 0.88), neurotoxicity (probability = 0.84). The cardiotoxicity, nutritional toxicity, and nephrotoxicity associated with oral GA were of particular concern, indicating that GA might lead to adverse cardiovascular events or kidney metabolism-related complications. 

### 3.5. Hypertension Key Effector Gene Screening

**Figure 6:**
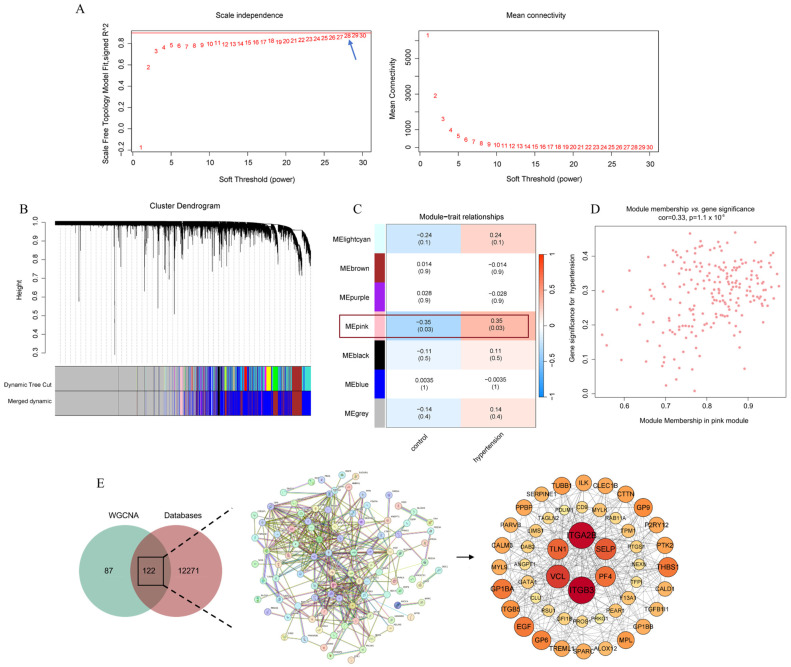
Network analysis and gene module selection related to hypertension. (**A**) Selection of the soft threshold power for WGCNA. (**B**) Screening of gene modules associated with hypertensive traits. (**C**) Gene modules showing high correlation with hypertension phenotypes. (**D**) Correlation plot of gene-trait relationships for the MEpink module. (**E**) Identification of hypertension-associated and effector genes.

Previous research has attributed the blood pressure fluctuations induced by GA to a pseudo-aldosteronism-like effect caused by GA and its metabolites. To explore other toxicological mechanisms by which GA influences blood pressure, this study aimed to identify potential hypertension effector genes. Transcriptomic data from hypertensive and control groups within the GSE71994 dataset were used to construct gene co-expression networks via the WGCNA method, identifying genes closely associated with the hypertension phenotype. An unscaled network was built with a correlation coefficient of 0.85 and a soft threshold of 28 (Figure 6A), and genes were clustered into different modules based on a cut height of 0.25 (Figure 6B). The MEpink module, with a strong correlation and statistical significance (cor = 0.33, *p* = 1.1 × 10^−6^), was selected for further analysis, consisting of 197 genes (Figure 6C,D).

Additionally, a total of 12,395 hypertension-related genes were retrieved from the GeneCards, OMIM, and TTD databases. The intersection of hypertension-related genes identified by both methods yielded 122 genes highly associated with hypertension. These 122 genes were imported into the STRING platform to construct a PPI network, and gene importance was ranked using Cytoscape software. The top 50 genes were selected as key candidates for subsequent analysis (Figure 6E).

### 3.6. Identification of Potential Hypertension Effector Genes

**Figure 7:**
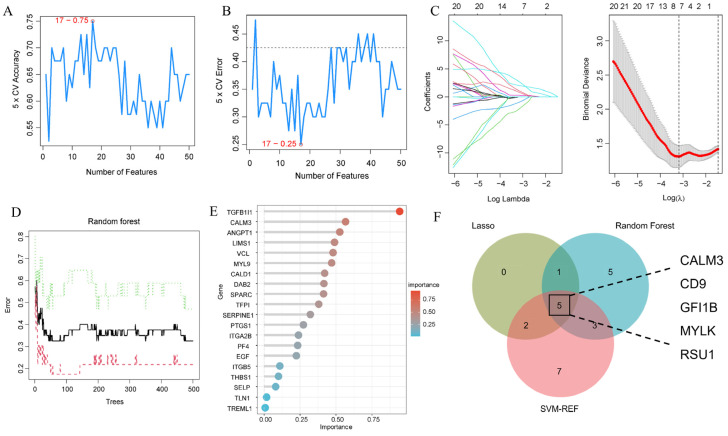
Identify key signature genes for hypertension by machine learning. (**A**,**B**) Accuracy and error rates of gene selection using the SVM-REF method. (**C**) Construction of the Lasso regression model. (**D**) Development of the Random Forest decision tree and identification of the point with minimal error. (**E**) Identification of hypertension-specific genes using the Random Forest approach. (**F**) Comparison of hypertension-specific gene selection across three machine learning algorithms.

The 50 candidate genes were further refined using machine learning algorithms to identify disease-specific biomarkers. The SVM-REF algorithm indicated that the classifier’s error was minimized when the number of features was 17 (Figure 7A,B). Lasso regression results showed that the optimal model fit was achieved with eight genes (Figure 7C). The Random Forests algorithm assigned importance scores to the 50 genes, and genes with an importance score > 0.5 were selected as disease-specific biomarkers (Figure 7D,E). The intersection of the three machine learning algorithms identified five genes as potential key biomarkers for hypertension: calmodulin 3 (CLAM3), CD9, growth factor independent 1B transcriptional repressor (GFI1B), myosin light chain kinase (MYLK), and Ras Suppressor Protein 1 (RSU1) (Figure 7F).

### 3.7. Validation of Hypertension Biomarker Predictive Probability

**Figure 8:**
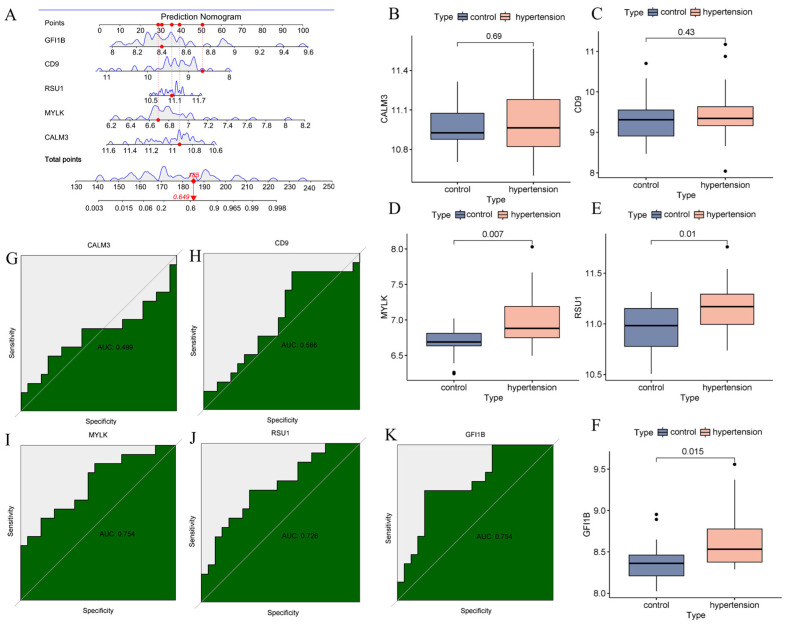
Validation of the diagnostic model for hypertension-specific signature genes. (**A**) Nomogram illustrating disease-associated genes. Expression levels of CALM3 (**B**), CD9 (**C**), MYLK (**D**), RSU1 (**E**), and GFI1B (**F**) in patients with hypertension. ROC curves for CALM3 (**G**), CD9 (**H**), MYLK (**I**), RSU1 (**J**), and GFI1B (**K**).

The nomogram suggested that the total score for the five genes was 185, yielding a moderate predictive probability (probability = 0.649) (Figure 8A). Expression levels of the five potential biomarkers in hypertensive patients were evaluated using GSE71994 data. GFI1B (*p* = 0.015), MYLK (*p* = 0.007), and RSU1 (*p* = 0.01) were significantly upregulated in hypertensive patients, whereas CALM3 (*p* = 0.69) and CD9 (*p* = 0.43) showed no statistically significant differences (Figure 8B–F). ROC curve analysis further revealed that the predictive accuracy of GFI1B (AUC = 0.754), MYLK (AUC = 0.7754), and RSU1 (AUC = 0.726) were high, while the models for CALM3 (AUC = 0.499) and CD9 (AUC = 0.586) had lower reliability (Figure 8G–K).

### 3.8. Mechanistic Exploration of GA-Induced Blood Pressure Fluctuations

**Figure 9:**
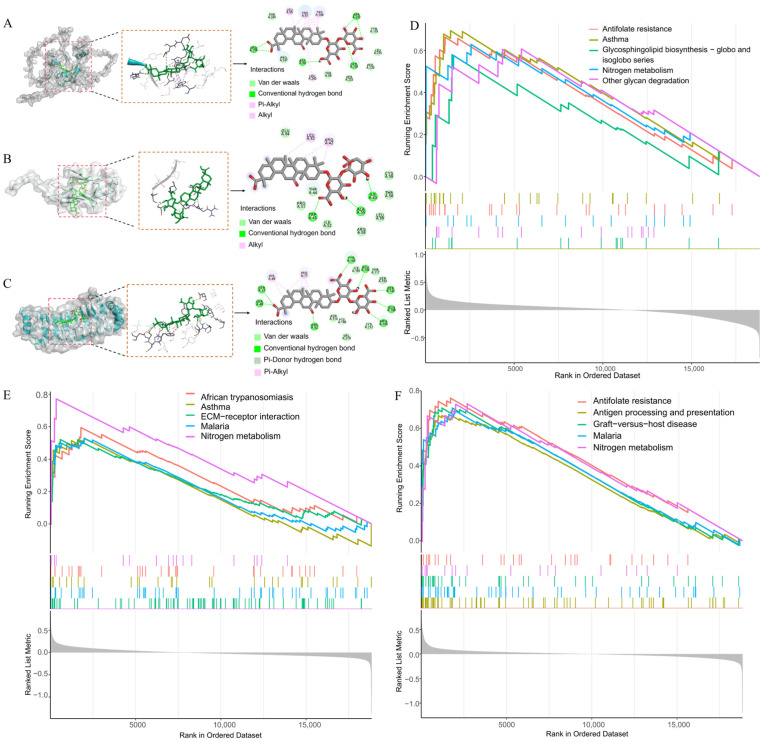
Investigation of the potential toxicological mechanisms of GA. Molecular docking results of GFI1B (**A**), MYLK (**B**), and RSU1 (**C**) with GA; GSEA results of GFI1B (**D**), MYLK (**E**), and RSU1 (**F**).

Based on the identified key biomarkers GFI1B, MYLK, and RSU1, we further explored the potential toxicological mechanisms of GA by conducting molecular docking and single-gene GSEA. Molecular docking results indicated that GA primarily binds to the GFI1B (Figure 9A), MYLK (Figure 9B), and RSU1 (Figure 9C) protein receptors through hydrogen bonding, van der Waals forces, hydrophobic interactions, and electrostatic interactions, with hydrogen bonds and van der Waals forces playing a dominant role. The binding energy values for all protein receptors exceeded 4.25 kcal/mol, suggesting stable binding (Supplementary Table S4). Given that GA can be metabolized into glycyrrhetinic acid in the gastrointestinal tract, subsequently entering the bloodstream to exert its effects, we also explored the binding affinity of glycyrrhetinic acid with GFI1B, MYLK, and RSU1 through molecular docking analysis. The results indicate a favorable binding interaction of glycyrrhetinic acid with GFI1B, MYLK, and RSU1 (Supplementary Table S5). Interestingly, the binding affinity trends of glycyrrhetinic acid with GFI1B, MYLK, and RSU1 mirror those of GA, with binding affinities ranked as GFI1B, RSU1, and MYLK in descending order. 

Given the elevated expression of GFI1B, MYLK, and RSU1 in hypertension, we performed GSEA to focus on their functional enrichment in this context. GSEA results revealed that these genes are involved in various pathophysiological processes, potentially underlying the toxicological mechanisms by which GA exerts its hypertensive effects (Figure 9D–F). Notably, the nitrogen metabolism pathway emerged as a common enriched pathway in the GSEA for GFI1B, MYLK, and RSU1, suggesting that GA may influence this pathway, thereby contributing to abnormal blood pressure fluctuations.

## 4. Discussion

Abnormal blood pressure fluctuations place a significant burden on organ systems and impair normal physiological functions [35]. Dietary factors can influence blood pressure through the induction of inflammatory responses or disruptions in electrolyte metabolism [36]. As a medicinal and edible plant, licorice has gained widespread attention due to its extensive use in both food and pharmaceuticals [6]. Previous studies have shown that licorice exerts its toxicological effects primarily through GA, which is metabolized into glycyrrhetinic acid in the intestines [37]. Glycyrrhetinic acid inhibits 11β-hydroxysteroid dehydrogenase, preventing the conversion of active cortisol into its inactive form [38]. This inhibition leads to elevated cortisol levels, which activate mineralocorticoid receptors, resulting in sodium and water retention as well as potassium excretion, ultimately causing hypertension and hypokalemia [39,40]. Additionally, Murck H et al. found that GA may influence the aldosterone/corticosterone ratio, thereby inhibiting the renin–angiotensin–aldosterone system and potentially inducing adverse effects [41]. The severity of these effects is dose-dependent and may vary based on individual metabolic responses. However, the primary compounds in licorice responsible for these hypertensive effects and their underlying toxicological mechanisms remain unclear. This study aimed to explore the impact of GA and LF on blood pressure fluctuations by conducting a literature review and network toxicology.

Meta-analysis revealed that GA intake significantly elevated both SBP and DBP, with a greater effect observed on SBP. In contrast, LF showed no significant impact on either SBP or DBP. Our findings suggested a relationship between GA and blood pressure, with high doses exerting a stronger hypertensive effect, particularly on SBP, which is consistent with the population study conducted by Af Geijerstam et al. [20]. Interestingly, in our subgroup analysis, we found that low doses of GA can increase blood pressure, while higher doses had no effect on DBP. Armanini et al. also observed no significant effect on DBP when subjects consumed approximately 300 mg of GA daily [42]. Their findings align with ours, although the underlying mechanism by which low-dose GA elevates DBP remains unclear. Moreover, given the limited number of studies included, the possibility of bias due to the available literature cannot be excluded. The World Health Organization recommends that GA intake should not exceed 100 mg/day; however, our results indicate that even lower doses carry a risk of raising blood pressure.

Given the evidence from the meta-analysis, GA is the key licorice component causing the increases in blood pressure. Using the Pro Tox-3.0 toxicological database, we identified that GA had potential oral toxicity and may induce adverse cardiovascular and renal events. Hypertension, a metabolic disorder, is not only linked to cardiovascular abnormalities but may also result from renal metabolic dysregulation [43,44]. Studies have shown that a prolonged intake of GA at concentrations of 0.1–1 mg/mL in drinking water can elevate right atrial pressure and pulmonary artery pressure in male Sprague Dawley rats. These rats exhibited mineralocorticoid excess syndrome, with systemic hypertension, water retention, hypernatremia, and hypokalemia [45]. In contrast, Watson et al. found no statistically significant blood pressure changes in dogs orally administered GA at doses of 0.1–0.6 mg/kg [46]. However, we observed an interesting phenomenon in their results: as the concentration of GA increased, the upper limit of systolic blood pressure in the dogs also tended to increase. In another study, continuous oral administration of licorice water extract to Wistar mice was found to induce high mineralocorticoid levels and lower cortisol and aldosterone concentrations [47]. These findings suggest the potential cardiovascular toxicity of GA, consistent with our toxicity predictions. Some studies also indicate that GA may protect myocardial function through anti-inflammatory or antioxidant pathways, suggesting that GA’s toxicity could be associated with microvascular damage without affecting the heart’s direct function [48]. Reports on GA nephrotoxicity are primarily case-based, including one of a 62-year-old woman with pre-existing conditions who developed acute kidney failure after consuming 200–250 g of black licorice daily [49], and a 48-year-old woman with anorexia nervosa who experienced acute kidney failure after ingesting 600 g of licorice within one hour. Currently, comprehensive in vivo studies on GA toxicity remain limited [50]. The hypertensive and other potential toxic effects of GA may be selective. A study by Somayeh Nazari et al. suggests that individuals with pre-existing conditions, elderly individuals, women, pregnant women, or infants may be more susceptible to GA-related adverse effects [51].

Historically, the hypertensive effects of licorice and its derivatives have been attributed to a pseudoaldosterone-like effect. In this study, we employed WGCNA and machine learning algorithms to identify potential biomarkers of hypertension to assess whether GA could induce blood pressure fluctuations through these targets. Initially, we identified 12,395 potential hypertension targets from online databases. To ensure a close association between the genes and the disease, we used WGCNA to refine this list to 209 genes closely related to the hypertension phenotype. Ultimately, we narrowed the pool to 122 genes using a combination of methods, which were further screened by machine learning to identify five key genes: GFI1B, MYLK, RSU1, CALM3, and CD9. However, we found that CALM3 and CD9 did not exhibit significant expression differences between the hypertension and control groups, and ROC analysis indicated low reliability for these genes as hypertensive biomarkers. Consequently, GFI1B, MYLK, and RSU1 were prioritized for further investigation.

GFI1B is a transcriptional repressor primarily expressed in hematopoietic and macrophage cells, known to be involved in hematopoietic disorders [52]. Research by Shooshtarizadeh et al. demonstrated that GFI1B forms a protein complex with β-catenin, modulating the activation of the Wnt/β-catenin signaling pathway, which is known to influence the renin-angiotensin system (RAS) and elevate blood pressure [53,54]. Although no direct evidence links GFI1B to blood pressure fluctuations, we hypothesize that it may regulate RAS activation via the Wnt/β-catenin pathway, leading to hypertension. Change in vascular tension is a key factor in blood pressure fluctuations, and MYLK, a kinase involved in myosin contraction and phosphorylation, plays a role in vasodilation at low expression levels [55,56]. In hypertensive patients, MYLK overexpression may induce smooth muscle contraction, leading to increased vascular resistance and blood pressure. RSU1, a Ras inhibitor, has been shown to activate ERK, promoting vascular remodeling and abnormal blood pressure regulation [57]. Together, these findings suggest that GFI1B, MYLK, and RSU1 may serve as potential hypertensive biomarkers, playing critical roles in the disease’s pathophysiological progression.

Previous perspectives have focused on the aldosterone-like effect of GA. Through the mechanisms, GA interferes with the RAS, indirectly suppressing renin release and activating the RAS pathway, ultimately contributing to elevated blood pressure. This study explores the role and potential mechanisms of GA on these biological factors through molecular docking after screening for new potential biomarkers of hypertension by machine learning. Our results show that GA formed stable complexes with GFI1B, MYLK, and RSU1 via hydrogen bonds and van der Waals forces, suggesting that GA’s hypertensive effects may be mediated through these genes. The molecular docking results indicated that GA forms stable complexes with GFI1B, MYLK, and RSU1 through hydrogen bonding and van der Waals forces. Additionally, GA showed binding energies with GFI1B, MYLK, and RSU1 exceeding −4.25 kcal/mol in absolute value [58]. As a secondary metabolite of GA, glycyrrhetinic acid plays a significant role in eliciting biological effects. Therefore, we also investigated the binding affinity of glycyrrhetinic acid with GFI1B, MYLK, and RSU1 through molecular docking, which similarly demonstrated stable binding. These docking results suggest that both GA and its metabolite glycyrrhetinic acid may interact with hypertension-related biomarkers. We previously discussed the relationship between GFI1B, MYLK, and RSU1 and hypertension, noting their roles in vascular resistance and renal metabolic pathways. This evidence supports the hypothesis that, beyond the traditional aldosterone-like pressor effect of GA and glycyrrhetinic acid, it may influence microvascular dynamics, altering blood flow resistance or leading to vascular remodeling, ultimately contributing to elevated blood pressure. GSEA, used to investigate gene–pathway associations, further substantiated our hypothesis in this study. Further, GSEA revealed that the high expression of GFI1B, MYLK, and RSU1 was associated with nitrogen metabolism pathways, which are linked to oxidative stress [59]. Dysregulation of nitrogen metabolism increases reactive nitrogen species, limiting nitric oxide (NO) bioavailability, which can lead to changes in vascular tone and microvascular damage, ultimately resulting in elevated blood pressure [60]. It was shown that licorice could attenuate vasodilation and increase resistance to blood flow via exogenous nitric oxide donors by Hautaniemi et al. [61]. This is consistent with the results of the present study.GA may therefore exert its hypertensive effects by modulating nitrogen metabolism through these key genes, subsequently impacting vascular resistance. 

However, during our quantitative analysis, we found that human intervention trials and animal studies specifically on GA monomers are limited. Given the widespread use of GA as a food sweetener, a tobacco processing aid, and in medicinal applications, we recommend future high-quality studies to better understand the long-term safety and potential toxicological mechanisms of GA or its salt compounds in specific populations.

## 5. Conclusions

In conclusion, our findings indicate that GA can elevate blood pressure, whereas LF has no significant effect on blood pressure. The potential hypertensive mechanism of GA and its metabolite glycyrrhetinic acid may involve interactions with GFI1B, MYLK, and RSU1, potentially disrupting nitrogen metabolism signaling pathways and contributing to vascular resistance changes. Based on these results, we suggest that individuals with hypertension or those susceptible to high blood pressure exercise caution when consuming GA-containing functional foods.

## Data Availability

All data are available with the consent of the corresponding author.

## Supplementary Materials

The following supporting information can be downloaded at https://www.mdpi.com/article/10.3390/nu16213768/s1, Figure S1: Analysis of bias by Cochrane handbook; Table S1: Literature search related to the intake of liquorice or liquorice components with blood pressure health; Table S2: Studies on the relationship between supplementation of glycyrrhizinic acid extracts and changes in blood pressure; Table S3: Meat regression and studies on the relationship between supplementation of licorice flavonoids extracts and changes in blood pressure; Table S4: Molecular docking binding energy of glycyrrhizinic acid; Table S5: Molecular docking binding energy of glycyrrhetinic acid.
