# Supplementary material for: Effects of Licorice Functional Components Intakes on Blood Pressure: A Systematic Review with Meta-Analysis and NETWORK Toxicology
Source: Nutrients. 2024 Nov 2;16(21):3768. doi: 10.3390/nu16213768 (PMC11547873; doi:10.3390/nu16213768)
Supplement: Supplementary file 1 [file nutrients-16-03768-s001.zip › Supplementary Figure S1 legend.pdf]

Supplementary Figure legend: Analysis of bias by cochrane handbook.
