# Supplementary material for: Effects of Licorice Functional Components Intakes on Blood Pressure: A Systematic Review with Meta-Analysis and NETWORK Toxicology
Source: Nutrients. 2024 Nov 2;16(21):3768. doi: 10.3390/nu16213768 (PMC11547873; doi:10.3390/nu16213768)
Supplement: Supplementary file 1 [file nutrients-16-03768-s001.zip › Supplementary Table S1.pdf]

Table S1. Literature search related to the intake of liquorice or liquorice components with blood pressure health.

Databasses	Search terms	Number of literature
Cochrane library	#1: (“Glycyrrhiza” OR "Licorice" OR " Licorices" OR “Liquorice” OR “Liquorices” OR “Glycyrrhiza glabra”) ti,ab,kw<br>#2: (“Blood pressure” OR " Pressure, Blood” OR “Pulse Pressure” OR “Pressure, Pulse” OR “Diastolic Pressure” OR “Pressure, Diastolic” OR “Systolic Pressure” OR “Pressures, Systolic” OR “Pressure, Systolic"):ti,ab,kw<br>#3: #1 AND #2	70
PubMed	(“Glycyrrhiza” OR "Licorice" OR " Licorices" OR “Liquorice” OR “Liquorices” OR “Glycyrrhiza glabra”) AND (“Blood pressure” OR " Pressure, Blood” OR “Pulse Pressure” OR “Pressure, Pulse” OR “Diastolic Pressure” OR “Pressure, Diastolic” OR “Systolic Pressure” OR “Pressures, Systolic” OR “Pressure, Systolic")	239
Medline	AB(Glycyrrhiza OR Licorice OR Licorices OR Liquorice OR Liquorices OR Glycyrrhiza glabra) AND AB(Blood pressure OR Pressure, Blood OR Pulse Pressure OR Pressure, Pulse OR Diastolic Pressure OR Pressure, Diastolic OR Systolic Pressure OR Pressures, Systolic OR Pressure, Systolic)	134
Embase	(licorice:ab,ti OR licorices:ab,ti OR liquorice:ab,ti OR liquorices:ab,ti OR 'glycyrrhiza glabra':ab,ti OR glycyrrhiza:ab,ti) AND ('pressure, blood':ab,ti OR 'pulse pressure':ab,ti OR 'pressure, pulse':ab,ti OR 'diastolic pressure':ab,ti OR 'pressure, diastolic':ab,ti OR 'systolic pressure':ab,ti OR 'pressures, systolic':ab,ti OR 'pressure, systolic':ab,ti OR 'blood pressure':ab,ti)	205
EBSCO	AB(Glycyrrhiza OR Licorice OR Licorices OR Liquorice OR Liquorices OR Glycyrrhiza glabra) AND AB(Blood pressure OR Pressure, Blood OR Pulse Pressure OR Pressure, Pulse OR Diastolic Pressure OR Pressure, Diastolic OR Systolic Pressure OR Pressures, Systolic OR Pressure, Systolic)	94
SCIENCE OF DIRECT	TI,AB,Keywords：（“Glycyrrhiza” OR "Licorice"）AND（“Blood pressure” OR " Pressure, Blood” OR “Pulse Pressure”）	40
WEB OF SCIENCE	((AB=(Licorice OR Licorices OR Liquorice OR Liquorices OR Glycyrrhiza glabra)) AND AB=(Pressure, Blood OR Pulse Pressure OR Pressure, Pulse OR Diastolic Pressure OR Pressure, Diastolic OR Systolic Pressure OR Pressures, Systolic OR Pressure, Systolic)) AND AB=(human)	156
**Total number**		938
