# Supplementary material for: Effects of Licorice Functional Components Intakes on Blood Pressure: A Systematic Review with Meta-Analysis and NETWORK Toxicology
Source: Nutrients. 2024 Nov 2;16(21):3768. doi: 10.3390/nu16213768 (PMC11547873; doi:10.3390/nu16213768)
Supplement: Supplementary file 1 [file nutrients-16-03768-s001.zip › Supplementary Table S2.pdf]

Table S2. Studies on the relationship between supplementation of glycyrrhizinic acid extracts and changes in blood pressure.

Author, year	Study type	Numbers	Basic information	Interventions	Resuts	Safety
CEM van Gelderen, 2000 a	RCT	19		The intervention group intake 1 mg/kg of glycyrrhetinic acid and the control group took placebo. Intervention for 8 weeks	No correlation was found between glycyrrhetinic acid intake and blood pressure	
CEM van Gelderen, 2000 b	RCT	19	Healthy female volunteers aged 19-40 years	The intervention group intake 2 mg/kg of glycyrrhetinic acid and the control group took placebo. Intervention for 8 weeks	Relatively elevated blood pressure compared to controls; no significant change in blood pressure compared to baseline	Two volunteers showed symptoms of hypokalaemia and difficulty concentrating
CEM van Gelderen, 2000 c	RCT	21		The intervention group intake 4 mg/kg of glycyrrhetinic acid and the control group took placebo. Intervention for 8 weeks	Relatively elevated blood pressure compared to controls; no significant change in blood pressure compared to baseline	
Miia H. Leskinen,2014	RCT	52	Healthy volunteers aged 25-42	The intervention group consumed 270-350 mg of liquorice per day, while the control group maintained a normal diet and did not consume liquorice-containing products	Increased systolic and diastolic blood pressure in the intervention group	No adverse reactions reported
Peder af Geijerstam, 2024	RCT	28	Healthy volunteers aged 18-30	The intervention group intake of the intervention containing 100 mg glycyrrhetinic acid; the control group intake of placebo	Increased blood pressure in young healthy participants with daily intake of glycyrrhizic acid containing 100 mg of glycyrrhizic acid	No adverse reactions reported
