# Supplementary material for: Effects of Licorice Functional Components Intakes on Blood Pressure: A Systematic Review with Meta-Analysis and NETWORK Toxicology
Source: Nutrients. 2024 Nov 2;16(21):3768. doi: 10.3390/nu16213768 (PMC11547873; doi:10.3390/nu16213768)
Supplement: Supplementary file 1 [file nutrients-16-03768-s001.zip › Supplementary Table S3.pdf]

Table S3-1. Meta-regression analysis of intervention dose and duration of intervention with SBP's WMD of LF.

Subgroup	Heterogeneity test(%)		Meta-regression analysis
	I^2	P value	
time	83.3	<0.001	0.335
dose			0.455

Table S3-2. Meta-regression analysis of intervention dose and duration of intervention with DBP's WMD of LF.

Subgroup	Heterogeneity test(%)		Meta-regression analysis
	I^2	P value	
time	83.3	<0.001	0.479
dose			0.479

Table S3-3. Studies on the relationship between supplementation of licorice flavonoids extracts and changes in blood pressure.

Author, year	Study type	Numbers	Basic information	Interventions	Resuts	Safety
Yacov Fogelman, 2016	RCT	110	Hyperlipidaemic volunteers aged 41-80 years	The intervention group ingested a daily ethanol extract of liquorice enriched with photoglycodine; the control group took a placebo. Intervention for 12 months	No correlation was found between liquorice extract intake and blood pressure	No adverse reactions reported
Sanjib Kumar Panda, 2017	RCT	50	Metabolic syndrome volunteers aged 18-75 years	The intervention group intake 300 mg/d of licorice flavonoid extract and the control group took placebo. Intervention for 12 weeks	Blood pressure in the intervention group did not show any significant change from baseline. There was no significant difference in systolic blood pressure between the intervention and placebo groups.	No adverse reactions reported
Zach W, 2011 a	RCT	22	Overweight volunteers aged 20-53 years with recreational activities	The intervention group intake 300 mg/d of licorice flavonoid oil extract and the control group took placebo. Intervention for 8 weeks	Licorice flavonoid oil intake has no significant effect on blood pressure	No adverse reactions reported
Zach W, 2011 b	RCT	23	Male athletes aged 19-35 years	The intervention group intake 300 mg/d of licorice flavonoid oil extract and the control group took placebo. Intervention for 8 weeks		
Yuji T ominag, 2009 a	RCT	39	Overweight volunteers between the ages of 40-60 years	The intervention group intake 300 mg/d of licorice flavonoid oil extract and the control group took placebo. Intervention for 8 weeks	No significant change in blood pressure compared to control group	No adverse reactions reported
Yuji T ominag, 2009 b	RCT	40		The intervention group intake 600 mg/d of licorice flavonoid oil extract and the control group took placebo. Intervention for 8 weeks		No adverse reactions reported
Yuji T ominag, 2009 c	RCT	40		The intervention group intake 900 mg/d of licorice flavonoid oil extract and the control group took placebo. Intervention for 8 weeks		No adverse reactions reported
Yuji T ominag, 2006 a	RCT	103	Moderately overweight volunteers aged 24-64 years	The intervention group intake 300 mg/d of licorice flavonoid oil extract and the control group took placebo. Intervention for 8 weeks	Licorice flavonoid oil has no significant effect on blood pressure changes	No adverse reactions reported
Yuji T ominaga, 2006 b	RCT	40		The intervention group intake 1800 mg/d of licorice flavonoid oil extract and the control group took placebo. Intervention for 8 weeks		Slight symptoms of malaise
