# Supplementary material for: Effects of Licorice Functional Components Intakes on Blood Pressure: A Systematic Review with Meta-Analysis and NETWORK Toxicology
Source: Nutrients. 2024 Nov 2;16(21):3768. doi: 10.3390/nu16213768 (PMC11547873; doi:10.3390/nu16213768)
Supplement: Supplementary file 1 [file nutrients-16-03768-s001.zip › Supplementary Table S4.pdf]

Table S4. Binding energy of molecular docking.

Target	Protein ID	compound	Binding energy(kcal/mol)
GFI1B	AF-Q5VTD9-F1-model_v4		-8.7
MYLK	2yr3	GA	-6.9
RSU1	7lt8		-7.8
